# Flavonol Glycosides: In Vitro Inhibition of DPPIV, Aldose Reductase and Combating Oxidative Stress are Potential Mechanisms for Mediating the Antidiabetic Activity of *Cleome droserifolia*

**DOI:** 10.3390/molecules25245864

**Published:** 2020-12-11

**Authors:** Amira Abdel Motaal, Heba H. Salem, Dalia Almaghaslah, Abdulrhman Alsayari, Abdullatif Bin Muhsinah, Mohammad Y. Alfaifi, Serag Eldin I. Elbehairi, Ali A. Shati, Hesham El-Askary

**Affiliations:** 1 Department of Pharmacognosy, College of Pharmacy, King Khalid University, Abha 61441, Saudi Arabia; alsayari@kku.edu.sa (A.A.); ajmohsnah@kku.edu.sa (A.B.M.); 2 Department of Pharmacognosy, Faculty of Pharmacy, Cairo University, Cairo 11562, Egypt; hesham.elaskary@pharma.cu.edu.eg; 3 Department of Biochemistry, Faculty of Pharmacy, Cairo University, Cairo 11562, Egypt; haminsalem@kku.edu.sa; 4 College of Pharmacy, King Khalid University, Abha 61441, Saudi Arabia; 5 Department of Clinical Pharmacy, College of Pharmacy, King Khalid University, Abha 61441, Saudi Arabia; damoazle@kku.edu.sa; 6 Department of Biology, Faculty of Science, King Khalid University, Abha 9004, Saudi Arabia; alfaifi@kku.edu.sa (M.Y.A.); serag@kku.edu.sa (S.E.I.E.); aaalshati@kku.edu.sa (A.A.S.); 7 Cell Culture Laboratory, Egyptian Organization for Biological Products and Vaccines, VACSERA Holding Company, Giza 22311, Egypt

**Keywords:** diabetes, aldose reductase, DPPIV, antioxidant, flavonols, *Cleome droserifolia*

## Abstract

Diabetes is a major health problem that is associated with high risk of various complications. Medicinal plants hold great promise against diabetes. The traditional use of *Cleome droserifolia* as an antidiabetic agent was correlated to its flavonol glycosides content. In the current study, five major flavonol glycosides appeared on the RP-HPLC chromatogram of the aqueous extract namely; quercetin-3-*O*-*β*-d-glucosyl-7-*O*-*α*-rhamnoside (**1**), isorhamnetin-7-*O*-*β*-neohesperidoside (**2**), isorhamnetin-3-*O*-*β*-d-glucoside (**3**) kaempferol-4′-methoxy-3,7-*O*-*α*-dirhamnoside (**4**), and isorhamnetin-3-*O*-*α*-(4″-acetylrhamnoside)-7-*O*-*α*-rhamnoside (**5**). The inhibitory activities of these compounds were tested in vitro against several enzymes involved in diabetes management. Only the relatively less polar methoxylated flavonol glycosides (**4**, **5**) showed mild to moderate α-amylase and α-glucosidase inhibitory activities. Compounds **1**–**4** displayed remarkable inhibition of dipeptidyl peptidase IV (DPPIV) enzyme (IC_50_ 0.194 ± 0.06, 0.573 ± 0.03, 0.345 ± 0.02 and 0.281 ± 0.05 µg/mL, respectively) comparable to vildagliptin (IC_50_ 0.154 ± 0.02 µg/mL). Moreover, these compounds showed high potential in preventing diabetes complications through inhibiting aldose reductase enzyme and combating oxidative stress. Both isorhamnetin glycoside derivatives (**2, 3**) exhibited the highest activities in aldose reductase inhibition and compound **2** (IC_50_ 5.45 ± 0.26 µg/mL) was even more potent than standard quercetin (IC_50_ 7.77 ± 0.43 µg/mL). Additionally, these flavonols exerted excellent antioxidant capacities through 2, 2-diphenyl-1-picrylhydrazil (DPPH) and ferric reducing antioxidant (FRAP) assays.

## 1. Introduction

Diabetes mellitus is a metabolic disorder characterized by hyperglycemia and associated with disorders in metabolism because of insufficient insulin required by the body [1]. Alpha-amylase and α-glucosidase are intestinal enzymes involved in the hydrolysis of starch into glucose and have a significant role in postprandial hyperglycemia. Hence, inhibition of these enzymes is crucial in the treatment of type 2 diabetes by delaying the digestion and absorption of disaccharides and starch [2]. Dipeptidyl peptidase IV (DPPIV) enzyme degrades the incretins, glucagon-like peptide 1 (GLP-1) and glucose-dependent insulinotropic polypeptide (GIP), that function by decreasing blood glucose through enhancement of insulin secretion and retardation of gastric emptying. Therefore, DPPIV inhibition has been used as an excellent target of new medications for type 2 diabetes [3]. Aldose reductase is an enzyme involved in catalyzing the reduction of glucose into sorbitol. During hyperglycemia associated with diabetes, significant quantities of sorbitol are produced due to the increased flux. Sorbitol accumulation participates in developing vascular and neurological complications of diabetes [4]. Thus, targeting this enzyme is of great benefit in the amelioration of diabetes complications.

Nowadays, the use of plant-derived natural products with therapeutic significance has attracted great attention due to their availability, affordability and relative safety. This motivates further search into traditional medicine to explore highly effective and safer natural anti-diabetic products that can probably hit several pathways. *Cleome droserifolia* (Forssk.) Del. herb (Cleomaceae) is known in the Middle East for diabetes treatment [5]. Various metabolites were isolated from the herb of this plant [6,7,8,9,10,11,12]. In addition, the antihyperglycemic activities of its different extracts were evaluated by several studies [8,13,14,15,16,17]. It was reported that *C. droserifolia* extract possessed a promising antidiabetic activity by a postprandial hypoglycemic effect and suppression to hepatic glucose output in the fasting state in albino rats. This was due to potentiation of peripheral and hepatic insulin sensitivity, and by diminishing intestinal glucose absorption [16]. The aqueous extract of *C. droserifolia*, standardized to contain not less than 1.5 ± 0.06% of kaempferol-4′-methoxy-3,7-dirhamnoside, showed a 63.3% activity of that of Metformin at 100 mg/kg body weight in rats, and raised the blood insulin level by 146.26% at this dose level [13].

We previously reported that the antidiabetic activity of the aqueous extract was higher than the ethanolic extract of *Cleome droserifolia* in cultured skeletal muscle cells and adipocytes. Treating muscle cells with the aqueous extract caused an increase in basal glucose uptake in a way comparable to insulin [6]. In another study, we demonstrated that the aqueous extract of *C. droserifolia* herb has a higher percent of the total flavonol glycosides (78%) compared to the ethanolic extracts. Furthermore, this aqueous extract was tested in vivo, at three different doses in alloxan-induced diabetic rats, where the highest dose (100 mg/kg) exhibited about 63% of the antihyperglycemic activity of metformin [13]. Therefore, the current study aimed at isolating the major compounds from the *C. droserifolia* aqueous extract in addition to investigating the possible mechanisms underlying the previously shown antidiabetic activities using several in vitro enzyme inhibition assays. Moreover, the antioxidant potential of the *C. droserifolia* extract and the isolated compounds was assessed. This could propose vital lead compounds for the treatment of diabetes and prevention from its complications. 

## 2. Results and Discussion

### 2.1. RP-HPLC Fingerprint Chromatogram of the Aqueous Extract

**Figure 1:**
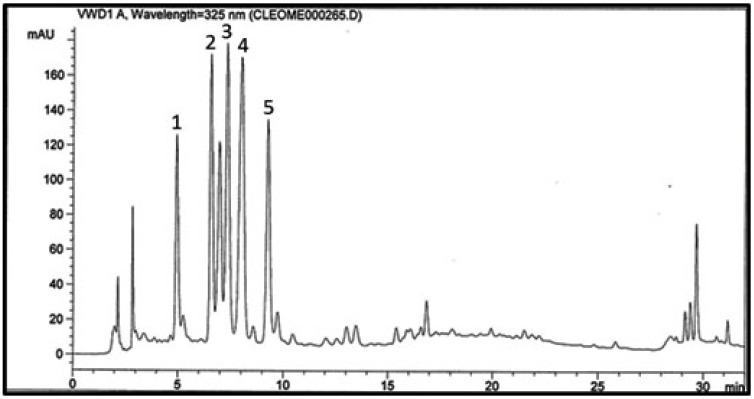
RP-HPLC fingerprint chromatogram of the aqueous extract of *C. droserifolia* showing peaks of major compounds: **1**, **2**, **3**, **4** and **5**.

A chromatographic profile was prepared for the aqueous extract of *C. droserifolia* showing five major peaks (Figure 1). Compounds **1** and **2** were eluted first at Rt 4.9 and 6.5, respectively, followed by compounds **3**, **4** and **5** at Rt 7.3, 8.0 and 9.2 min, respectively. This required the isolation of these major five compounds to explore their possible mechanisms of action, and to determine their contribution to the folk and previously proven antidiabetic activity of the holistic aqueous extract [6,13].

### 2.2. Structural Elucidation of the Isolated Compounds

**Figure 2:**
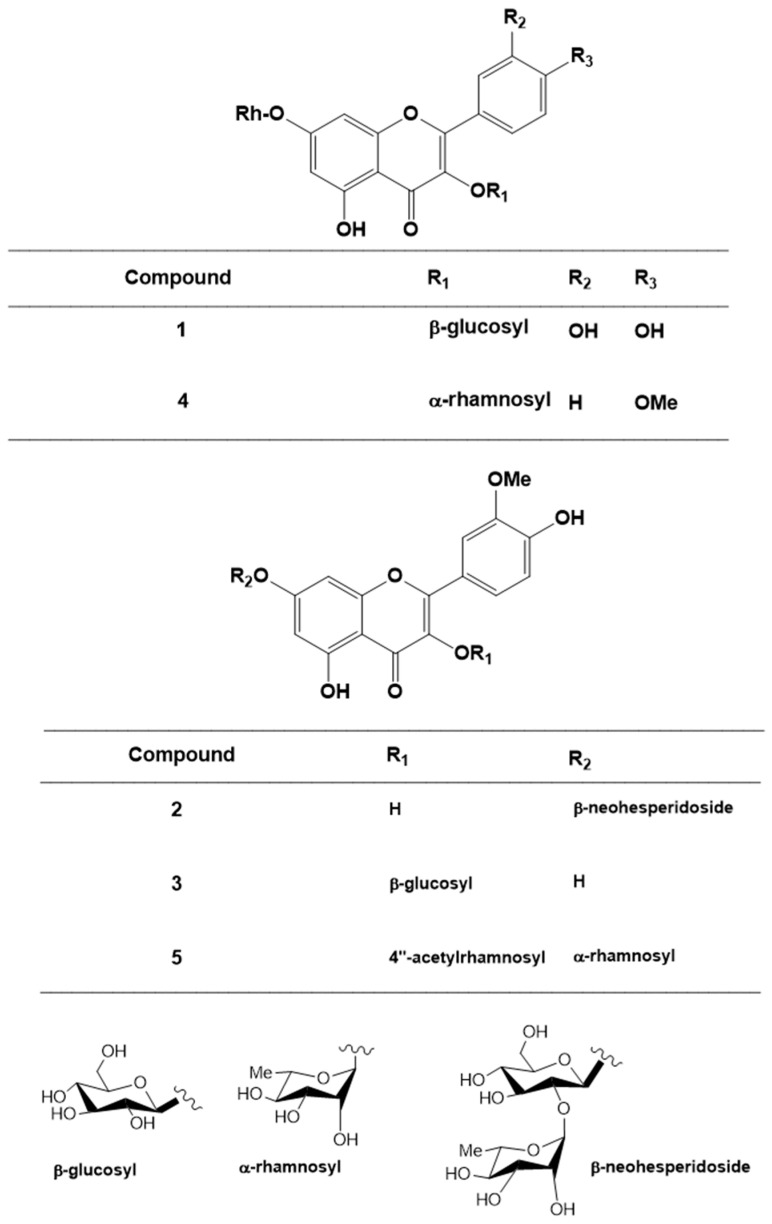
Chemical structures of compounds **1**–**5**.

Compound **1** was yellow amorphous powder; mp 195 °C. The ^1^H and ^13^C-NMR data showed the characteristic signals for a quercetin nucleus (Table 1 and Table 2). The glycosidic characters in C-3 and C-7 positions were confirmed by the two anomeric protons (δ_H_ 5.3 and 5.58 ppm; δ_C_ 102.52 and 99.2 ppm) for glucose and rhamnose moieties, respectively, and the doublet (δ_H_ 1.27 ppm; δ_C_ 16.68 ppm) which was assigned to the methyl group of the rhamnose. All data matched those of a flavonol glycoside previously isolated from *C. droserifolia* [9,17]. Compound **1** was therefore identified as quercetin-3-*O*-*β*-d-glucosyl-7-*O*-*α*-rhamnoside (Figure 2).

Compound **2** was yellow amorphous powder; mp 225 °C. Two meta-coupled doublets at δ_H_ 6.46 and 6.86 (*J* = 2.0 Hz) correlated to two methines at δ_C_ 99.85 and 95.05 and were assigned to C-6 and C-8, respectively (Table 1 and Table 2). A methoxyl signal appeared at δ_H_ 3.59 and two sugars were indicated from the two anomeric protons at δ_H_ 5.58 and 5.56 ppm. The chemical shifts of the sugar carbons were in complete agreement with those reported for 2-*O*-*α*-l-rhamnopyranosyl-*β*-d-glucopyranose (neohesperidose) [7]. All data were consistent with a flavonol glycoside which was isolated from *C. droserifolia* [7]. Thus, compound **2** was identified as isorhamnetin-7-*O*-*β*-d-neohesperidoside (Figure 2).

Compounds **3** and **4** were isolated as yellow crystalline powder; m.p. 243 and 247 °C, respectively. Their ^1^H and ^13^C-NMR data were in agreement with those reported for isorhamnetin-3-*O*-*β*-d-glucoside and kaempferol-4′-methoxy-3,7-*O*-*α*-dirhamnoside, respectively (Figure 2) previously isolated from *C. droserifolia* [11].

Compound **5** was yellow crystalline powder; m.p. 257 °C. Its ^1^H and ^13^C-NMR spectra (Table 1 and Table 2) showed the characteristic signals for a quercetin nucleus and a methoxy group at δ_H_ 3.87 ppm and δ_C_ 55.7 ppm. Its position was also confirmed by the upfield shift of C-2′ (δ_C_ 113.1 ppm). Two anomeric protons (δ_H_ 5.55 and 5.31 ppm; δ_C_ 101.3 and 99.4 ppm) appeared as singlets and two doublets (δ_H_ 1.13 and 0.82 ppm; δ_C_ 17.4 and 17.1 ppm) were assigned to the two methyl groups of rhamnose moieties attached to 3-OH and 7-OH, respectively. An acetyl group appeared as (3H, s) at δ_H_ 2.00 ppm, δ_C_ 20.9 and 169.9 ppm (OCOCH3). All spectral data matched a flavonol glycoside previously isolated from *C. droserifolia* by members of our group [11]. Thus compound **5** was identified as isorhamnetin-3-*O*-*α*-(4″- acetylrhamnoside)-7-*O*-*α*-rhamnoside (Figure 2).

### 2.3. Intestinal Enzymes Inhibition

**Figure 3:**
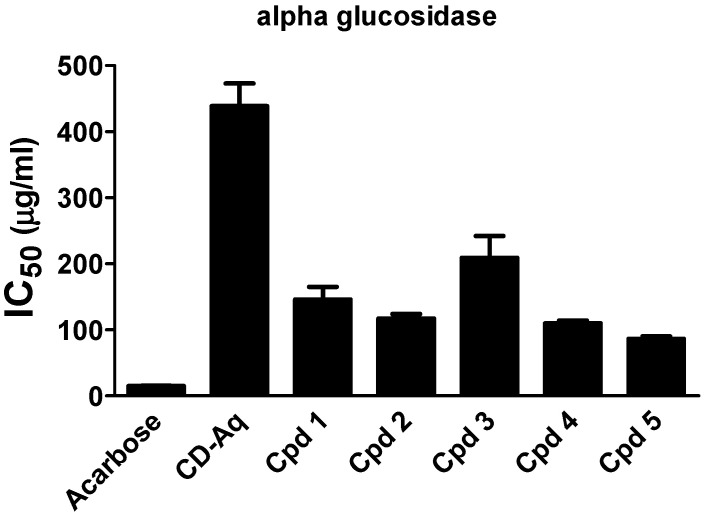
Alpha glucosidase inhibitory activity of the aqueous extract of *Cleome droserifolia* and the isolated compounds (**1**–**5**). Values are shown as mean ± SEM of two replicates. CD-Aq, aqueous extract; Cpd, compound.

Several enzymes are involved in diabetes management. Two of these enzymes are the intestinal enzymes α-amylase and α-glucosidase that are involved in the hydrolysis of starch into glucose. Thus, inhibition of these enzymes has been identified as a potentially important approach in the management of diabetes by controlling the postprandial hyperglycemia and retarding glucose absorption [4]. In this study, the in vitro α-amylase inhibition of the aqueous extract of *C. droserifolia* along with compounds **1**–**5** were evaluated. Only compounds **4** and **5** possessed mild α-amylase inhibitory activity with IC_50_ values of 152.6 ± 35.75 µg/mL and 106.3 ± 2.50 µg/mL, respectively, compared to acarbose reference standard (IC_50_ = 0.57 ± 0.04 µg/mL). No inhibitory activity was observed by the *C. droserifolia* extract nor compounds **1**–**3**. On the other hand, the α-glucosidase enzyme inhibition assay declared that although the aqueous extract of *C. droserifolia* exerted a weak inhibitory activity, all isolated compounds showed moderate dose-dependent inhibitory activities. Among these, compounds **5** and **4** displayed the most prominent α-glucosidase inhibitory activities with IC_50_ values of 86.54 ± 3.8 and 110.09 ± 4.2 µg/mL, respectively (Figure 3), compared to 14.81 ± 0.4 µg/mL of acarbose.

### 2.4. Dipeptidyl Peptidase IV (DPPIV) Inhibition

**Figure 4:**
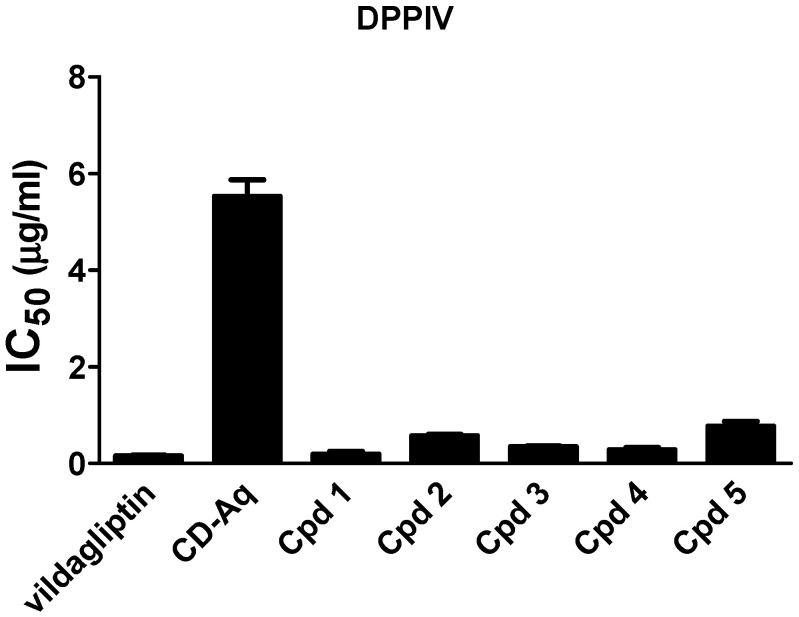
Dipeptidyl peptidase IV (DPPIV) inhibitory activity of the aqueous extract of *Cleome droserifolia* and the isolated compounds (**1**–**5**). Values are shown as mean ± SEM of two replicates. CD-Aq, aqueous extract; Cpd, compound.

Dipeptidyl peptidase IV (DPPIV) enzyme inactivates the incretins, GLP-1 and GIP. Thus, glucose tolerance in diabetes patients is improved through inhibiting DPPIV by enhancing glucose-dependent insulin release in addition to stimulating β-cell proliferation [3]. The inhibition potential of the *C. droserifolia* extract and isolated compounds was measured in vitro as depicted in Figure 4. Results revealed that the extract and the isolated compounds exerted high inhibitory activities. Of notice, the isolated compounds (**1**–**4**) had IC_50_ values of 0.194 ± 0.06, 0.573 ± 0.03, 0.345 ± 0.02 and 0.281 ± 0.05 µg/mL that were comparable to the commercially available DPPIV inhibitor, vildagliptin (IC_50_ 0.154 ± 0.02 µg/mL).

### 2.5. Aldose Reductase Inhibition

**Figure 5:**
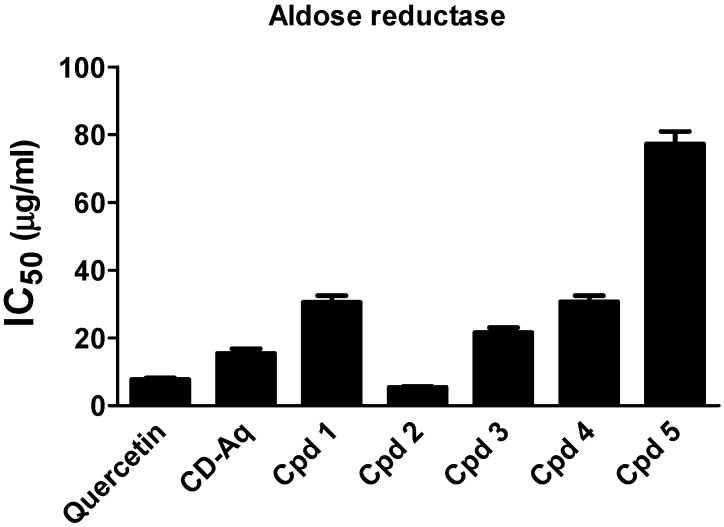
Aldose reductase inhibitory activity of the aqueous extract of *Cleome droserifolia* and the isolated compounds (**1**–**5**). Values are shown as mean ± SEM of two replicates. CD-Aq, aqueous extract; Cpd, compound.

The aldose reductase enzyme is crucial in the development of vascular and neurological complications of diabetes [4]. Thereby targeting this enzyme is of great value in treating diabetes complications such as neuropathy, nephropathy and retinopathy. The incubation of either the aqueous extract of *C. droserifolia* or the isolated compounds (**1**–**5**) with aldose reductase enzyme induced a remarkable decrease in its activity (Figure 5). The IC_50_ values were recorded at 30.51 ± 1.95, 5.45 ± 0.26, 21.55 ± 1.52, 30.68 ± 1.82 and 77.24 ± 3.71 µg/mL for the *C. droserifolia* isolated compounds (**1**–**5**), respectively. Interestingly, the reference standard quercetin (IC_50_ 7.77 ± 0.43 µg/mL) possessed only 70% of the activity of compound **2**. 

From the above results, it could be deduced that the folk antidiabetic potential of *C. droserifolia* aqueous extract is possibly conveyed through its major constituents of flavonol glycosides (78%) attaining different mechanisms of action. Where the two relatively less polar methoxylated flavonol glycosides (**4** and **5**) contributed partly by moderately inhibiting the intestinal enzymes (α-amylase and α-glucosidase) and thus could retard glucose absorption. Intriguingly, these two compounds showed before significant insulin-like effects in vitro by increasing basal glucose uptake [6]. Moreover, members of our research group proved previously that these compounds possessed potent cytotoxic activities against breast (MCF7), colon (HCT116), and liver (HepG2) carcinoma, where compound (**5**) harnessed HepG2 tumor progression in a TP53/miR-15/miR-16 dependent manner [11,18].

However, the five major flavonol glycosides could potentially mediate the antidiabetic activity of the aqueous extract through the observed remarkable inhibition of DPPIV enzyme, which was comparable to the commercial drug vildagliptin. Thus, flavonol glycosides of *C. droserifolia* could be considered as potential lead compounds for type 2 diabetes mellitus treatment possibly by prolonging the incretins actions in stimulating insulin release, augmenting β-cell proliferation and combating hyperglycemia. Previous literature reported the in vitro DPPIV inhibitory activity of flavonol glycosides (kaempferol glycosides) found in *Lens culinaris* [3]. Further studies also proved the in vivo DPPIV inhibitory activity of flavonoids in serum of type 2 diabetic mice [19] and normal albino rats [20], as well as their ability to increase serum GLP1 level [19]. Here we report DPPIV inhibition by kaempferol, quercetin and isorhamnetin glycosides. This might explain, at least in part, the previously reported antihyperglycemic effect of kaempferol-3,7-*O*-*α*-dirhamnoside [21], isorhamnetin-3-*O-β*-d-glucoside [22] and quercetin [23] in diabetic rats.

Moreover, the aqueous extract of *C. droserifolia* possessed a potential ability in preventing diabetes complications as conveyed by the strong aldose reductase inhibitory activity of its flavonol glycosides (**1**–**5**), in particular isorhamnetin-7-*O*-*β*-d-neohesperidoside (**2**) and isorhamnetin-3-*O*-*β*-d-glucoside (**3**). Notably, compound **2** was more potent than the reference standard quercetin. While isorhamnetin-3-*O*-α-(4″-acetylrhamnoside)-7-*O*-α-rhamnoside (**5**), showed the least aldose reductase inhibitory activity as indicated in Figure 5, which may be due to presence of two *α*-rhamnosyl sugars at position 3 and 7 of the isorhamnetin nucleus. While the sugar moieties in compound **2** is *β*-d-neohesperidosyl at position 7 only, and in compound **3** is *β*-d-glucosyl at position 3 only of the isorhamnetin nucleus. It was previously described that isorhamnetin-3-*O*-*β*-d-glucoside isolated from *Salicornia herbacea* inhibited aldose reductase and sorbitol accumulation in streptozotocin-induced diabetic rat tissues [22]. In agreement, both quercetin and naringin (a flavonoid glycoside) have shown inhibitory effects on lens aldose reductase of healthy and diabetic rats [24]. It was also reported that flavonoid glycosides especially glucosides were more efficiently absorbed from the small intestine than from the colon which led to higher plasma levels [25].

### 2.6. In Vitro Antioxidant Activity

**Figure 6:**
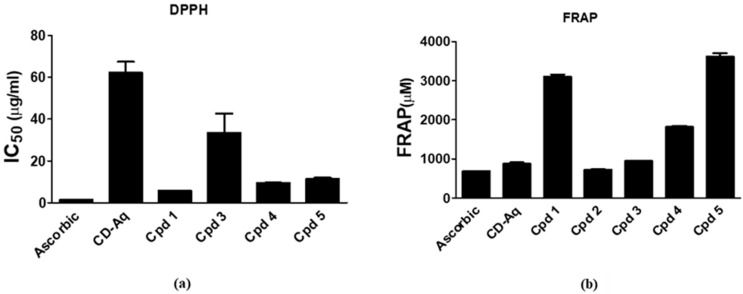
Antioxidant activity of the aqueous extract and isolated compounds from *Cleome droserifolia*. (**a**) DPPH radical scavenging. (**b**) Ferric reducing antioxidant power (FRAP). Values are shown as mean ± SEM of two replicates. CD-Aq, aqueous extract; Cpd, compound; DPPH, 2,2-diphenyl-1-picrylhydrazyl.

Increased oxidative stress is involved in the pathogenesis of diabetes mellitus complications [26]. In the current study, the antioxidant capacity of the aqueous extract of *C. droserifolia* and its isolated flavonol glycosides was investigated using the 2, 2-Diphenyl-1-picrylhydrazil (DPPH) and ferric reducing antioxidant power (FRAP) assays. Tested samples exerted excellent free radical scavenging activities as measured by the DPPH method. Of these, compounds **1**, **4**, **5** exhibited the highest activities with IC_50_ values attained at 5.67 ± 0.09, 9.44 ± 0.13 and 11.45 ± 0.48 µg/mL, respectively (Figure 6a). The standard ascorbic acid had an IC_50_ of 1.37 ± 0.07 µg/mL. Strikingly, results of the FRAP assay declared that all compounds together with the aqueous extract exhibited very potent ferric reducing activity even exceeding that of ascorbic acid. In consistence with the DPPH assay, compounds **5**, **1**, and **4** possessed the highest activities with FRAP values of 3610 ± 88.3, 3106 ± 52.2 and 1812 ± 33.2 µM, respectively, as compared to 685.2 ± 1.07 µM for the ascorbic acid (Figure 6b). Antioxidant effects of flavonol glycosides from different plants have been previously demonstrated [27,28]. Thus, these flavonol glycosides might contribute to the antidiabetic potential of *C. droserifolia* aqueous extract being powerful antioxidants and could offer a protective effect against diabetes complications by mitigating the oxidative stress.

## 3. Materials and Methods

### 3.1. Plant Material

The herb of *Cleome droserifolia* (Forssk.) Delile, Cleomaceae, was obtained from Sinai in 2015. Dr. M. Gebali (National Research Center, Egypt) authenticated the plant. A voucher specimen (No. 22.4.2015) was kept at the herbarium of the Pharmacognosy Department, Faculty of Pharmacy, Cairo University.

### 3.2. General

Alpha-glucosidase enzyme from *Saccharomyces cerevisiae*, α-amylase from porcine pancreas and 2-chloro-4-nitrophenyl-α-D-maltotrioside (CNPG3) were purchased from Sigma Aldrich. All other chemicals were purchased from UFC Biotechnology. A microplate reader (Anthos Zenyth-200RT, Cambridge, UK) was used for the spectrophotometric measurements. A Bruker NMR Spectrometer (Bruker, Yokohama, Japan) used for ^1^H-NMR (400 MH_Z_) and ^13^C-NMR (100 MHz) analysis. Spectra were recorded in CD_3_OD or DMSO-d_6_ using tetramethylsilane (Sigma-Aldrich, St. Louis, MO, USA) as an internal standard, and chemical shift values were expressed in δ ppm.

### 3.3. Extraction and Fractionation

The dried powdered herb of *C. droserifolia* (200 g) was extracted with boiled water (3 × 400 mL) by sonication at 20 °C for 5 min. It was allowed to stand overnight for 3 days, sonicated again for 5 min and filtered. The aqueous extract was concentrated and partitioned with methylene chloride (4 × 100 mL). It was then fractionated with H_2_O and methanol (RP-18-VLC column, 50 g, 8 × 4 cm). Fractions (100 mL) were collected and monitored by HPLC. Compounds **1**–**5** were eluted with 25–27%, 35–40%, 40–50%, 50–50% and 45–55% methanol/H_2_O, respectively. The 5 compounds were further purified yielding 145, 210, 195, 230 and 130 mg of compounds **1**–**5**, respectively.

### 3.4. HPLC Analysis of the Aqueous Extract

An Agilent technologies 1100 series HPLC system equipped with an Agilent 1200 series quaternary pump (Agilent Technologies, Santa Clara, CA, USA), a degasser G1322A and a UV detector at λ_max_ 325nm was used. Data acquisition was accomplished using Agilent ChemStation software (Agilent Technologies, Santa Clara, CA, USA). The aqueous extract sample (1 mg/mL methanol) was injected (20 μL, injection volume) into a LiChrosphere 100 RP-C18 column (5 μm, 250 mm L × 4 mm D, Merck, Germany) maintained at room temperature, preceded by RP-C18 guard column (5 μm, 10 mm L × 4 mm D). Acetonitrile (solvent A) and 0.3% *O*-phosphoric acid in water (solvent B) were used as mobile phase. A continuous gradient elution (10–75% A in B) for 25 min was carried out at a flow rate of 1.0 mL/min.

### 3.5. In Vitro Antidiabetic Activity

#### 3.5.1. α-Amylase Inhibition Assay

Inhibition of the aqueous extract and the isolated compounds on the α-amylase enzyme activity was determined kinetically using acarbose as a standard [29]. A pre-incubation mixture was prepared which contained 40 mM phosphate buffer (pH 6.9) together with different concentrations (10, 50, 100, 200 µg/mL) and (5, 20, 50, 100 µg/mL) for the parent extract and the isolated compounds, respectively. A 0.2 U of α-amylase enzyme was added to this mixture and the plate was then incubated for 15 min at 37 °C. Then, 2-chloro-4-nitrophenyl maltotrioside (CNPG3) substrate was added where the final concentration was 0.9 µmole/mL. The increase in absorbance at 405 nm, which is proportional to the enzyme activity, was recorded for 3 min. A control which was devoid of the test samples was also run in parallel. The 50% inhibitory concentration (IC_50_) was determined and presented for the parent extract, the isolated compounds and the standard.

#### 3.5.2. α-Glucosidase Inhibition Assay

The activity of the parent extract of *C. droserifolia* together with the isolated compounds was assessed spectrophotometrically according to Li et al. (2009) [30] with slight modifications. In a volume of 100 µL of 40 mM phosphate buffer (pH 6.9), different concentrations (10, 50, 100, 200 µg/mL) for the parent extract and (5, 20, 50, 100 µg/mL) for the isolated compounds were prepared. The tested samples were added to a reaction mixture in a 96-well microplate containing 20 µL of the phosphate buffer and 20 µL of 5 mM p-nitrophenyl α -D-glucopyranoside (PNP-G) in the buffer. Then 20 µL of 0.4 U/mL α-glucosidase in the buffer were added and the plate was mixed and incubated at 37 °C for 15 min. After incubation, the absorbance was measured at 405 nm in the microplate reader. Acarbose was used as a reference compound and a control was performed without the test samples. The IC_50_ for test compounds as well as the standard was calculated and presented.

#### 3.5.3. Dipeptidyl Peptidase IV (DPPIV) Inhibition Assay

The DPPIV Inhibitor Screening Kit (Abnova, Taipei, Taiwan) was used in accordance to the manufacturer׳s protocol. In this assay, DPPIV cleaved a substrate to produce a quenched fluorescent group (Em/Ex = 360/460 nm). This cleavage was abolished in presence of a DPPIV inhibitor [3]. Vildagliptin was also run as a positive control inhibitor. To a 50 μL DPPIV Enzyme Solution, we added 25 μL of DPP4 Assay Buffer, vildagliptin or different concentrations of either the parent extract (10, 50, 100, 200 µg/mL) or the isolated compounds (5, 20, 50, 100 µg/mL). The plate was then incubated at 37 °C for 10 min after which 25 μL DPPIV Substrate Solution was added and measurement was done at Ex/Em = 360/460 nm. Another measurement was recorded after further incubating the reaction at 37 °C for 15–30 min and the difference between the two readings was calculated. The IC_50_ was determined and presented for the parent extract, the isolated compounds and the standard.

#### 3.5.4. Aldose Reductase Inhibition Assay

Determination of the aldose reductase inhibitory effectiveness of the investigated samples was performed kinetically using the Aldose Reductase Inhibitor Screening Kit (Biovision, Milpitas, CA, USA), according to the manufacturer׳s protocol. The decrease in the absorption of NADPH at 340 nm was measured in a mixture containing NADPH, the enzyme and its substrate, DL-glyceraldeyde in addition to test samples [31]. Different concentrations of the parent extract (10, 50, 100, 200 µg/mL) and of the isolated compounds (5, 20, 50, 100 µg/mL) were used and quercetin was the reference standard in the assay. Briefly, 60 μL of NADPH was added to all wells containing 10 μL of either the Enzyme Assay Buffer, test samples or quercetin. A 90 μL of freshly prepared Aldose Reductase Solution was then added and the plate was incubated protected from light at 37 °C for 15–20 min. After incubation, 40 μL enzyme substrate in assay buffer was added and the absorbance was measured at 340 nm kinetically for 60–90 min at 37 °C. A background control free from the enzyme was run and subtracted from the readings. The IC_50_ was calculated and presented for the samples and the standard.

### 3.6. In Vitro Antioxidant Activity

#### 3.6.1. 2,2-diphenyl-1-picrylhydrazil (DPPH) Radical Scavenging Assay

The free radical scavenging capacity of the parent extract of *C. droserifolia* together with the isolated compounds was estimated using 2,2-diphenyl-1-picrylhydrazil (DPPH) as described previously [32]. In brief, 240 µL of 0.004% DPPH in methanol was added to 8 µL of the parent extract and the isolated compounds at different concentrations: (2, 4, 12, 16 µg/mL) and (0.5, 1, 3, 4 µg/mL), respectively. The reaction mixture was mixed and incubated in dark for 30 min at room temperature. The absorbance was then measured at 517 nm against blank that was done using water instead of the plant extract. Ascorbic acid was used as the antioxidant standard and the IC_50_ value for samples and standard was determined.

#### 3.6.2. Ferric Reducing Antioxidant Power (FRAP) Assay

The antioxidant potential of the extract and the isolated compounds was assessed according to the assay originally designed by Benzie and Strain (1996) [33] using the DetectX^®^ Ferric Reducing Antioxidant Power (FRAP™) Detection Kit by Arbor assays, Ann Arbor, USA. In the provided plate, 75 µL of the prepared FRAP Color Solution was mixed with 20 µL of either the aqueous extract (400 µg/mL), the isolated compounds (200 µg/mL), different concentrations of the standard ferrous chloride or ascorbic acid positive control. The mixture was incubated for 30 min at room temperature and the produced blue colored product was read at 560 nm. The FRAP values were calculated and expressed in µM FeCl_2_.

## 4. Conclusion

The aqueous extract of *C. droserifolia* is traditionally used to treat hyperglycemia. It showed antidiabetic potential in several in vitro and in vivo studies. The RP-HPLC finger-print chromatogram of the aqueous extract showed five major peaks for flavonol glycosides which constituted 78% of the extract. In the current study, in vitro experiments proved that these five flavonol glycosides could inhibit DPPIV enzyme responsible for enhancing the insulin-releasing effects of incretins, in addition to inhibiting the intestinal enzymes involved in the hydrolysis of polysaccharides into glucose. These effects might, at least in part, represent possible mechanisms for the previously reported antidiabetic activity of *C. droserifolia*. Furthermore, these flavonol glycosides could offer protection against diabetes complications by virtue of inhibiting aldose reductase enzyme, and ameliorating the oxidative stress known to be involved in the pathogenesis of diabetes complications. However further studies are required to confirm their in vivo efficacies and to explore other potential mechanisms.

## Figures and Tables

**Table 1:** ^13^C-NMR chemical shifts (δ in ppm) for compounds **1**–**5** (CD_3_OD or DMSO-d_6_, 100 MHz).

C	1	2	3	4	5
2	158.07	157.28	156.3	156.0	156.0
3	134.38	133.75	132.7	134.5	134.4
4	178.23	178.05	177.1	177.7	177.8
5	161.38	161.34	161.1	160.1	161.8
6	98.45	99.85	98.8	98.5	98.4
7	162.17	162.05	165.1	161.6	160.4
8	94.09	95.05	93.8	94.5	94.6
9	156.57	156.45	156.9	157.7	157.6
10	105.97	106.14	103.6	105.8	105.7
1′	121.50	121.40	121.4	120.2	120.4
2′	114.67	113.92	113.4	130.5	113.1
3′	144.55	147.4	146.7	115.3	147.4
4′	148.63	150.06	149.3	160.8	149.8
5′	116.16	115.7	115.1	115.3	115.5
6′	121.96	122.75	121.9	130.5	120.7
1″	102.52	101.18	100.8	101.8	101.3
2″	76.67	76.87	74.3	70.2	71.7
3″	74.33	74.79	76.4	70.5	70.1
4″	69.86	70.29	69.8	71.6	69.8
5″	77.02	77.96	77.3	70.0	68.0
6″	61.14	61.09	60.5	17.8	17.4
1‴	99.20	98.78		99.3	99.4
2‴	70.28	70.5		70.2	70.3
3‴	70.67	70.53		70.3	69.9
4‴	72.20	72.07		71.1	73.2
5‴	69.86	70.29		69.7	67.5
6‴	16.68	18.38		17.3	17.1
OCH_3_-3′			55.6		55.7
OCH_3_-4′				55.7	
OCOCH_3_					169.9
OCOCH_3_					20.9

**Table 2:** ^1^H-NMR chemical shifts (δ ppm) for compounds **1**–**5** (CD_3_OD or DMSO-d_6_, 400 MHz, *J* in Hz).

H	1	2	3	4	5
6	6.47 1H, d, *J* = 1.8	6.46 1H, d, *J* = 2.0	6.18 1H, d, *J* = 1.2	6.44 1H, d, *J* = 1.2	6.45 1H, d, *J* = 1.2
8	6.75 1H, br.s	6.86 1H, d, *J* = 2.0	6.41 1H, d, *J* = 1.2	6.77 1H, d, *J* = 1.2	6.78 1H, d, *J* = 1.2
2′	7.74 1H, d, *J* = 1.8	7.96 1H, d, *J* = 2.0	7.94 1H, br.s	7.77 2H, d, *J* = 8.4	7.45 1H, br.s
3′	-	-	-	6.92 2H, d, *J* = 8.4	-
5′	6.89 1H, d, *J* = 8.5	6.93 1H, d, *J* = 8.4	6.89 1H, d, J = 8.4	6.92 2H, d, *J* = 8.4	6.93 1H, d, *J* = 8.4
6′	7.6 1H, dd, *J* = 1.8, 8.5	7.57 1H, dd, *J* = 2.1,8.5	7.48 1H, dd, *J* = 2.1,8.4	7.78 2H, d, *J* = 8.4	7.44 1H, dd, *J* = 2.1,8.4
1″	5.3 1H, d, *J* = 7.5	5.58 1H, d, *J* = 7.5	5.54 1H, d, *J* = 6.9	5.55 1H, s	5.55 1H, s
4″	-	-	-	-	4.72 1H, t
6″	-	3.59 1H, dd, *J* = 7.0, 11	-	1.13 3H, d, *J* = 6	1.13 3H, d, *J* = 6
1‴	5.58 1H, s	5.56 1H, br. s	-	5.31 1H, s	5.31 1H, s
6‴	1.27 3H, d, *J* = 6	0.82 3H, d, *J* = 6	-	0.82 3H, d, *J* = 6	0.82 3H, d, *J* = 6
OCH_3_-3′	-	3.86 3H, s	3.83 3H, s	-	3.87 3H, s
OCH_3_-4′			-	3.86 3H, s	-
OCOCH_3_			-	-	2.00 3H, s

## Abbreviations

*C. droserifolia*	Cleome droserifolia
CNPG3	2-chloro-4-nitrophenyl -α-d-maltotrioside
DPPIV	Dipeptidyl peptidase IV
DPPH	2, 2-Diphenyl-1-picrylhydrazil
FRAP	Ferric reducing antioxidant power
GIP	Glucose-dependent insulinotropic polypeptide
GLP-1	Glucagon-like peptide 1
PNP-G	p-Nitrophenyl α -d-glucopyranoside
